# Systemic α-synuclein injection triggers selective neuronal pathology as seen in patients with Parkinson’s disease

**DOI:** 10.1038/s41380-019-0608-9

**Published:** 2019-11-22

**Authors:** Wei-Li Kuan, Katherine Stott, Xiaoling He, Tobias C. Wood, Sujeong Yang, Jessica C. F. Kwok, Katie Hall, Yanyan Zhao, Ole Tietz, Franklin I. Aigbirhio, Anthony C. Vernon, Roger A. Barker

**Affiliations:** 1 John van Geest Centre for Brain Repair, Department of Clinical Neuroscience, University of Cambridge, Cambridge, CB2 0PY UK; 2 Department of Biochemistry, University of Cambridge, Cambridge, CB2 1GA UK; 3 Department of Neuroimaging, Kings College London, London, SE5 8AF UK; 4 School of Biomedical Sciences, Faculty of Biological Sciences, University of Leeds, Leeds, LS2 9JT UK; 5 Centre for Reconstructive Neuroscience, Institute of Experimental Medicine, 142 20 Prague 4, Czech Republic; 6 Wolfson Brain Imaging Centre, Department of Clinical Neurosciences, University of Cambridge, Cambridge, CB2 0QQ UK; 7 Department of Basic and Clinical Neuroscience, Institute of Psychiatry, Psychology and Neuroscience, King’s College London, London, SE5 9RX UK; 8 MRC Centre for Neurodevelopmental Disorders, King’s College London, London, SE1 1UL UK; 9 Department of Neurology, Addenbrooke’s Hospital, Cambridge, CB2 0QQ UK; 10 Wellcome Trust—MRC Cambridge Stem Cell Centre, Cambridge, CB2 1QR UK

**Keywords:** Biological techniques, Neuroscience

## Abstract

Parkinson’s disease (PD) is an α-synucleinopathy characterized by the progressive loss of specific neuronal populations. Here, we develop a novel approach to transvascularly deliver proteins of complex quaternary structures, including α-synuclein preformed fibrils (pff). We show that a single systemic administration of α-synuclein pff triggers pathological transformation of endogenous α-synuclein in non-transgenic rats, which leads to neurodegeneration in discrete brain regions. Specifically, pff-exposed animals displayed a progressive deterioration in gastrointestinal and olfactory functions, which corresponded with the presence of cellular pathology in the central and enteric nervous systems. The α-synuclein pathology generated was both time dependent and region specific. Interestingly, the most significant neuropathological changes were observed in those brain regions affected in the early stages of PD. Our data therefore demonstrate for the first time that a single, transvascular administration of α-synuclein pff can lead to selective regional neuropathology resembling the premotor stage of idiopathic PD. Furthermore, this novel delivery approach could also be used to deliver a range of other pathogenic, as well as therapeutic, protein cargos transvascularly to the brain.

## Introduction

Parkinson’s disease (PD) is a progressive neurological disorder defined by the loss of nigrostriatal dopaminergic neurons and formation of α-synuclein-positive Lewy bodies (LB) [1]. It is well established that neurodegeneration and LB pathology affects only a subset of vulnerable cells in PD. Recent evidence, however, suggests that pathogenic α-synuclein can propagate between cells and seed pathology in a prion-like manner [2, 3]. The question therefore arises as to why such a spread only generates pathology in certain neuron populations. Indeed, while neuropathological studies suggest that there is a stereotypic, spatiotemporal pattern of LB deposition in post-mortem PD brains [4, 5], studies using a direct intracerebral injection of α-synuclein preformed fibrils (pffs) to transform endogenous α-synuclein into pathological species are dependent on the site of injection [6–9]. This approach therefore does not capture the pathology of PD nor does it reflect the experimental evidence suggesting that α-synuclein pathology can spread from the periphery, via the enteric (ENS), to the central nervous system (CNS) [10].

Misfolded α-synuclein has been shown to cross the blood–brain barrier following repeated systemic administration, possibly through downregulating endothelial tight-junction proteins, but at a level that does not generate α-synuclein pathology or neurodegeneration [11, 12]. We therefore sought to optimize the efficiency of this transvascular protein transduction by taking advantage of a modified rabies virus glycoprotein (RVG9R), which has previously been used to mediate transvascular RNA delivery to the brain [13, 14]. Negatively charged nucleotides can be electrostatically complexed with a cationic nona-arginine sequence, while the RVG ligand itself interacts with a neural-specific acetylcholine receptor to induce a transient membrane inversion and enable translocation of the RNA cargo into the cytoplasm [15]. By interacting with anionic amino acids on the surface of proteins, we hypothesized that RVG9R could also be used to enable efficient protein transduction into the CNS following peripheral administration, without it affecting the physiological activities of the cargo protein.

Using this approach, we demonstrate for the first time that systemic delivery of pathological α-synuclein pffs using this method can lead to selective pathology, neurodegeneration, and functional deficits in the rat CNS and ENS. Critically, this occurs in a pattern that strikingly mirrors that reported in early-stage, idiopathic PD patients. This model therefore recapitulates some key features of early-stage PD, and so has significant potential for pre-clinical testing of putative disease-modifying agents, when such agents are thought to have their maximal chance of therapeutic benefit. Finally, the strategy we used for transvascular delivery of α-synuclein could also be leveraged to deliver other pathogenic and/or therapeutic proteins to the CNS.

## Materials and methods

### Peptide/protein complex formation

To prepare the complex for delivery, peptides and protein cargos were mixed in PBS in a glass vial (Supelco) and left on an orbital shaker (Stuart SSM1, 60 rpm), for 30 min at room temperature before use. The optimal (peptide:cargo protein) molar ratio for application was empirically determined to be 10:1 for monomeric proteins. The molecular weights for the heterodimeric chondroitinase ABC (ChABC) and misfolded α-synuclein pff were assumed to be 120 and 14.5 kDa, respectively, and the peptide/protein complex was mixed at a 10:1 molar ratio.

### Animal procedures

Female Sprague-Dawley (SD) rats, weighing around 200 g at the time of injection, were housed with unrestricted access to food and water unless otherwise stated. Animals were housed in groups of four on a 12 hours light/dark cycle. For intravenous delivery, a total volume of 300 μl of the peptide/protein complex, containing 100 μg of the cargo proteins or 3 U ChABC per animal, was administered via the lateral tail vein.

A detailed “Material and methods” section can be found in the Supplementary information.

## Results

### The RVG9R system mediates efficient protein transduction

To ascertain if the RVG9R system could mediate protein transduction in vitro, monomeric human α-synuclein or the green fluorescent protein (GFP) were added in culture alone or mixed with RVG9R at 1:10 molar ratio. Overnight incubation of RVG9R:α-synuclein or RVG9R:GFP with primary rat or human foetal cortical neurons, respectively, induced cellular update of the cargo proteins (Supplementary Fig. 1a). Adult SD rats were then intravenously administered with either RVG9R:α-synuclein, RVG9R:GFP, or appropriate RVG9R or cargo controls and, after 24 hours, distinctive axonal human-specific α-synuclein and somatic GFP stainings were detectable in various CNS regions (Supplementary Fig. 1b). To assess the biodistribution of RVG9R-mediated cargo delivery, animals were given a single intravenous RVG9R:GFP administration and, after 24 hours, GFP expression was detectable in the CNS only when complexed with RVG9R (Supplementary Fig. 1c). The presence of GFP in the peripheral organs of RVG9R:GFP injected animals could be attributed to the rabies virus-mediated anterograde trans-synaptic from the CNS to the peripheral nerve terminals [16], although further investigation is required to validate whether RVG9R behaves in a similar fashion as the intact virus.

We next assessed whether the RVG9R system can transvascularly deliver biologically active proteins, such as the heterodimeric ChABC enzyme. The formation of chondroitin sulfate proteoglycans-enriched perineuronal nets (PNNs), which are abundantly expressed in the sensorimotor cortex, impedes CNS plasticity, and this can be modulated by enzymatic digestion of chondroitin sulfate chains using ChABC [17]. A single peripheral injection of RVG9R:ChABC treatment progressively reduced *Wisteria floribunda* agglutinin (WFA)-positive PNNs. Consistently the expression of unsulfated C-0-S stubs, resulting from ChABC digestion, was also elevated, although the level of extracellular cartilage link protein 1 remained unaffected (Supplementary Fig. 1d, e). This suggested that RVG9R-delivered ChABC removed chondroitin sulfates without affecting PNN structure.

### RVG9R reversibly complexes with the C-terminus of α-synuclein

**Fig. 1:**
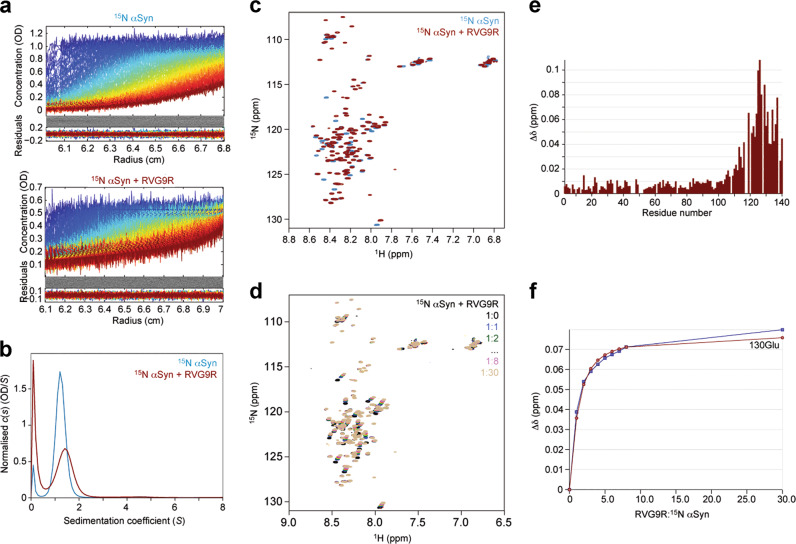
RVG9R interacts with the C-terminus of α-synuclein. **a** AUC sedimentation velocity data for α-synuclein alone, and in the presence RVG9R (both 30 μM). **b** α-Synuclein was monomeric with an *s*^o^_20,w_ value of 1.2 S, assuming a uniform frictional ratio of *F*_k,w_ ~ 1.9. The value of *s*^o^_20,w_ increased to 1.4 S in the presence of RVG9R, indicative of complex formation. Free RVG9R was observed close to *s* = 0. The final root-mean-square deviation was 0.04 in each case. **c** HSQC of ^15^N α-synuclein with a saturating level of RVG9R (30:1 molar ratio), and **d** chemical-shift difference vs residue number. **e** HSQC of ^15^N α-synuclein with increasing concentrations of RVG9R. **f** Representative data showing *K*_d_ extraction from ∆δ of Glu130 using a 1:1 binding model

To further understand how RVG9R complexes with proteins, we first studied the interaction between RVG9R and α-synuclein using analytical ultracentrifugation (AUC). Recombinant human α-synuclein was observed to be monomeric but, in the presence of RVG9R, a shift of the sedimentation coefficient was observed, indicative of complex formation (Fig. 1a, b). The interaction was studied in detail by NMR spectroscopy using ^15^N-labelled α-synuclein, for which chemical-shift assignments were available [18]. RVG9R was titrated into ^15^N-labelled α-synuclein and the chemical-shift changes monitored by ^15^N heteronuclear single quantum coherence (HSQC) spectroscopy, until saturation was achieved (Fig. 1c). By far the most significant chemical-shift changes were observed for residues 106–140 at the C-terminus of α-synuclein, which is both anionic and disordered (Fig. 1d). The shift changes for the peaks showing the largest changes were fitted to a one-site model to calculate affinity (Fig. 1e, f). Equilibrium was achieved within 2 min of the addition of RVG9R for each titration point. An average dissociation constant (*K*_d_) of 80 ± 4 µM for RVG9R:α-synuclein was calculated from the fitted *K*_d_ for residues 120–140 at the C-terminus, leading to a lower limit of *k*_off_ > 0.004 s^−1^, corresponding to a dissociative half-life (*ln*2/*k*_off_) of <170 s (Fig. 1f). As *k*_off_ is independent of concentration, this would be expected to hold true in vivo.

### A single intravenous administration of RVG9R:α-synuclein pff generates GI and olfactory deficits

**Fig. 2:**
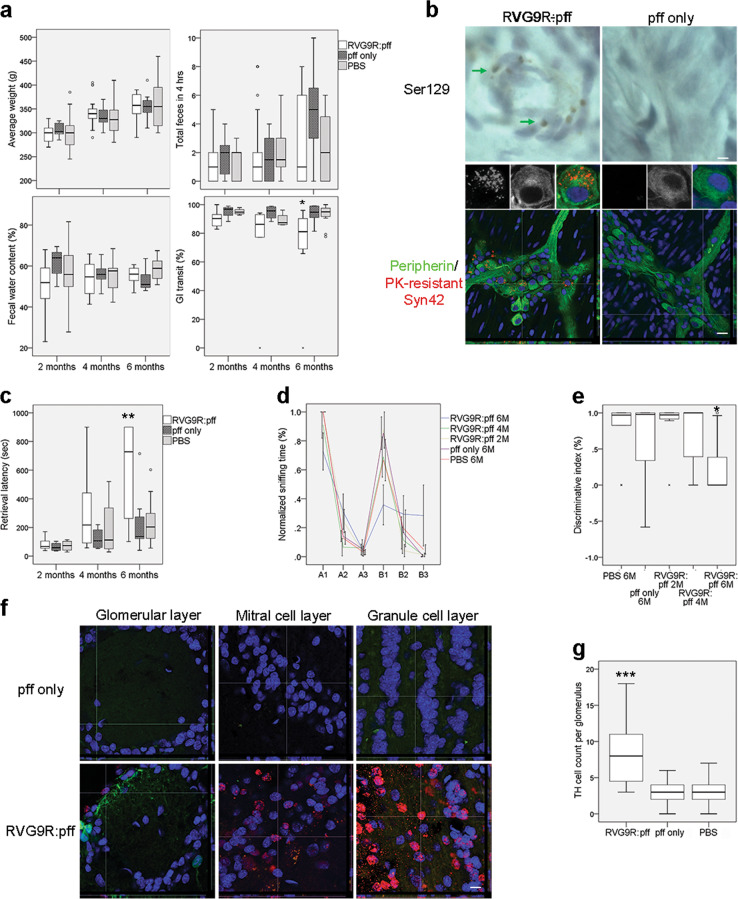
Transvascular RVG9R:pff delivery generates GI and olfactory impairments. **a** No group differences were observed in body weight and faecal profiles over time, although there was a treatment effect in GI transit (*F*_2,55_ = 4.559, *p* = 0.015), reaching statistical significance at 6 months after a single intravenous RVG9R:pff injection (*p* = 0.021 vs pff alone, *p* = 0.028 vs PBS). **b** Abnormally phosphorylated (DAB-peroxidase, green arrows) and PK-resistant α-synuclein inclusions (red) were detected in the duodenal myenteric plexus (peripherin, green). **c** There was a significant treatment effect in the latency to retrieve food at 6 months post injection (*F*_2,54_ = 4.483, *p* = 0.016), post hoc analysis vs pff alone (*p* = 0.002) and PBS (*p* = 0.003) groups. **d** There was a trend for the RVG9R:pff treated rats to be less capable at discriminating the new odour presentation (A3-B1) at 6 months post injection, although this failed to reach significance after post hoc adjustment. **e** There was a significant reduction on the discrimination index in the RVG9R:pff rats at 6 months post injection (*F*_4,43_ = 2.710, *p* = 0.042), post hoc analysis vs PBS at 6 months post injection (*p* = 0.041) and RVG9R:pff at 2 months post injection (*p* = 0.022). **f** Extensive α-synuclein phosphorylation (Ser129, red) was found in the mitral and granule cell layers, with more TH^+^ neurons (green) also being observed in the periglomerular region in the RVG9R:pff rats. **g** There was a significant increase in the TH cell counts per glomerulus in the RVG9R:pff rats at 6 months post injection (*F*_2,33_ = 39.843, *p* < 0.001), post hoc analysis vs pff only (*p* < 0.001) and PBS (*p* < 0.001) groups. *p < 0.05, **p < 0.01, ***p < 0.001 compared with PBS group at the same time point. Data were analyzed using multivariate ANOVA or repeated measure ANOVA in **d**. *n* = 6–8 per group at 2/4 months post injection, *n* = 9–12 at 6 months post injection. Three glomeruli were randomly selected for analysis from each animal and averaged in **g**. Nuclei were counterstained with Hoechst (blue). Scale bars, 30 µm

It was recently shown that full-length human α-synuclein pffs have a highly ordered core with unstructured tails [19]. We therefore hypothesized that this C-terminus region could be accessible to RVG9R complexing and sought to investigate whether RVG9R-mediated delivery of wild-type human pff (Supplementary Fig. 2a) can induce α-synuclein pathology when delivered non-selectively to the nervous system. Rats were given a single intravenous administration of either RVG9R:pff, pff alone, or PBS, and followed behaviourally over a 6-month period. Animals receiving RVG9R alone were not included as our previous work has shown that repeated administration of the RVG9R control did not generate any phenotypes [14]. Over the assessment period, there were no significant group differences in their locomotor function, motor coordination, and affective behaviour (Supplementary Fig. 3). There were also no significant group differences in body weight gain or in faecal profiles over the 6-month period. In contrast, solid GI transit was significantly prolonged at 6 months post injection, specifically in the RVG9R:pff cohort, suggestive of subtle GI dysfunction (Fig. 2a). Post-mortem analysis in these animals revealed phosphorylated α-synuclein inclusions and PK-resistant aggregates in the myenteric plexus, but not in the submucosal plexus, of the duodenum (Fig. 2b, Supplementary Fig. 2b). Whilst this was not accompanied by overt cell loss or changes in total α-synuclein expression in the duodenum (which exists in dimeric forms [20]), there was a significant increase in glial fibrillary acidic protein (GFAP) expression in the LMMP, as well as an elevation in GFAP^+^ reactive astrocytes around the myenteric plexus (Supplementary Fig. 2c, d).

Together with GI dysfunction, hyposmia is another clinical feature of premotor PD. We therefore assessed the ability of the RVG9R:pff exposed animals to use olfactory cues for foraging after overnight fasting. In the buried food test, the RVG9R:pff animals displayed an increasing retrieval latency over time (Fig. 2c). Similarly, odour-cross habituation was disrupted in these animals at 6 months post injection, with a significant group effect on the discrimination index (Fig. 2d, e). Post-mortem histopathological analysis of the olfactory bulb (OB) revealed substantial phosphorylation of α-synuclein in the mitral and granule, but not in the glomerular cell layer, in RVG9R:pff animals (Fig. 2f). Using the novel pentameric thiophene derivative (pFTAA) that specifically recognizes oligomeric and fibrillar protein species [21], we observed no evidence of abnormal protein folding in these regions (Supplementary Fig. 2e). There was however a significant elevation in the number of dopaminergic cells residing locally in the periglomerular area (Fig. 2f, g), although the level of tyrosine hydroxylase (TH) expression and the concentration of dopamine in the whole OB remained unchanged (Supplementary Fig. 2f–h).

### Structural imaging changes on MRI and their functional correlates

**Fig. 3:**
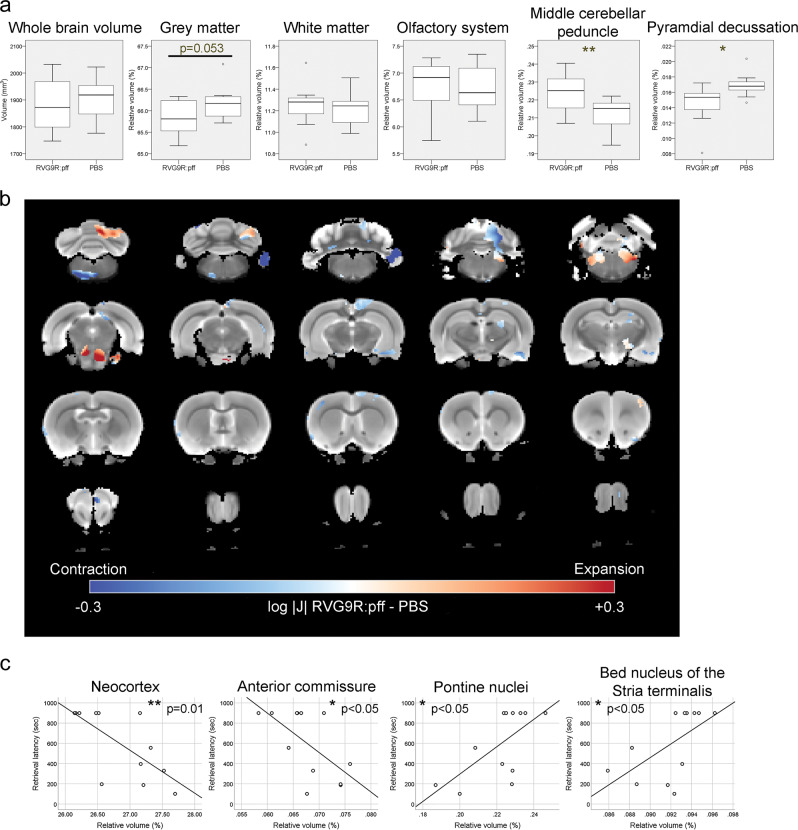
RVG9R:pff treatment induces subtle neuroanatomical changes on MRI at 6 months post injection. **a** Data shown are the relative volume differences by region, derived from ABS of MR images. No changes survived adjustment for multiple comparisons (5% false discovery rate, FDR). A trend (*F*_1,22_ = 4.188, *p* = 0.053) for global grey matter atrophy was observed in the RVG9R:pff treated rats. Increased relative volume of the MCP (+5%, *F*_1,22_ = 8.693, *p* = 0.007, uncorrected *q* = 0.33) and a decreased relative volume of PDec (−14%, *F*_1,22_ = 8.470, *p* = 0.008, uncorrected *q* = 0.33), were also observed in RVG9R:pff rats. **b** At an exploratory threshold (*p* < 0.05 uncorrected), RVG9R:pff exposure induced subtle effects on local volume and revealed previously unappreciated clusters of voxels with volumetric changes in the brain stem and cerebellum. **c** Linear regression revealed significant correlations between the relative volumes of four brain regions (*r*^2^ = 0.50 for neocortex, *r*^2^ = 0.39 for anterior commissure, *r*^2^ = 0.43 for pontine nuclei, and *r*^2^ = 0.36 for BNST) and retrieval latency in the buried food test in RVG9R:pff treated rats. Associated *p* values (uncorrected) are shown. *p < 0.05, **p < 0.01. Data were analyzed using *t*-tests for each atlas ROI and then corrected using FDR. Data shown in **b** are the results of statistical testing for differences in the log scaled Jacobean determinant between RVG9R:pff and PBS-injected rats, at an exploratory threshold of *p* < 0.05 uncorrected for multiple comparisons. *n* = 12 per group

We next examined if there were any macroscale changes in rat brain volume following RVG9R:pff exposure and if so, how these related to the subtle behavioural phenotype of these animals. Comparing the relative volumes of the summary regions of interest (ROIs) for total grey and white matter and the individual rat brain ROIs (*n* = 79), there were no significant treatment effects after multiple comparison adjustment (Fig. 3a). The relative volumes of the olfactory system were also not affected, despite the presence of olfactory deficits (Fig. 3a). The relative volumes of two individual atlas ROIs in the RVG9R:pff rats, the medial cerebellar peduncle (MCP) and the pyramidal decussation (PDec), were increased (+5) and decreased (−14), respectively (Fig. 3a). Effect sizes (Cohen’s *d*) for each region were +1.3 and −1.3, respectively. Whole-brain, voxel-wise TBM analysis confirmed these data, with no significant volumetric changes between treatment groups after stringent correction for multiple comparisons (family-wise error rate, *p* < 0.05). There were however clusters of voxels with both apparent changes in volume diffusely in the neocortex, brain stem, and cerebellum, at an exploratory threshold of *p* < 0.05 (uncorrected, Fig. 3b).

We next explored our MRI dataset for correlations between relative brain ROI volumes in the RVG9R:pff treated animals with their performance in the buried food test. Since this is an exploratory analysis we did not correct the resulting *p* values for multiple comparisons. This analysis suggested that two ROIs, the neocortex and anterior commissure, were negatively correlated with olfactory impairment, such that smaller relative volumes of these structures were associated with longer retrieval latency (Fig. 3c). A further 2/80 ROIs, the pontine nuclei and the bed nucleus of the stria terminalis (BNST), were positively correlated with performance on the buried food test (Fig. 3c). There were no such correlations with any of these regions in the control group.

### RVG9R:pff treatment induces neurodegeneration in the midbrain and brain stem

**Fig. 4:**
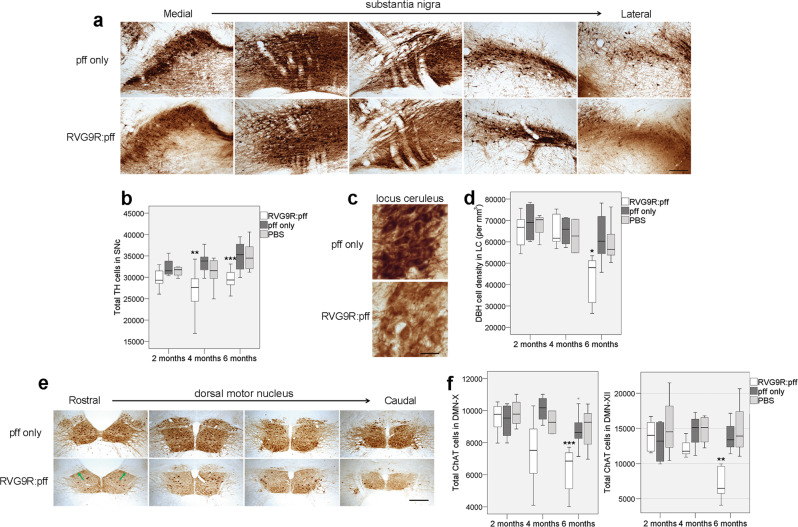
A single intravenous injection of RVG9R:pff induces progressive CNS pathology in non-transgenic rats. **a** Representative pictures of the TH^+^ dopaminergic neurons in the midbrain of adult rats. **b** There was a significant treatment effect on TH^+^ cell counts in the SNc (*F*_2,71_ = 16.242, *p* < 0.001), post hoc analysis revealed a significant dopaminergic neurodegeneration vs pff alone (*p* = 0.01) at 4 months post injection, and vs pff alone (*p* < 0.001) and PBS (*p* < 0.001) groups at 6 months post injection. **c** Representative pictures of the DBH^+^ noradrenergic neurons in the LC. **d** There was a significant treatment effect on DBH^+^ cell numbers per mm^3^ in the LC (*F*_2,45_ = 4.708, *p* = 0.014), post hoc analysis revealed a significant noradrenergic neurodegeneration vs pff alone (*p* = 0.002) and PBS (*p* = 0.012) groups at 6 months post lesion. **e** Representative pictures of the ChAT^+^ cholinergic neurons in the DMN-X (green arrows) and DMN-XII. **f** There was a significant treatment effect over time on ChAT^+^ cell counts in the DMN-X (*F*_4,53_ = 2.799, *p* = 0.035), as well as in the DMN-XII (*F*_4,53_ = 3.005, *p* = 0.033), post hoc analysis revealed a significant cholinergic neurodegeneration in the DMN-X vs pff alone (*p* < 0.001) and PBS (*p* < 0.001) groups, as well as in the DMN-XII vs pff alone (*p* < 0.01) and PBS (*p* < 0.01) groups, at 6 months post lesion. *p < 0.05, **p < 0.01, ***p < 0.001 compared with pff only group at the same time point. Data were analyzed using multivariate ANOVA, *n* = 6–12 per group at 2 and 4 months post injection, *n* = 8–12 at 6 months post injection. Scale bars, 500 µm for **a** and **e**, and 100 µm for **c**

We next sought to define whether there was any relevant CNS histopathology in brain regions reported to show neuropathology in early-stage PD [4, 5], as well as relevant sites affected at trend level in the MRI data. There were significant reductions of TH^+^ and vesicular monoamine transporter-positive (VMAT2^+^) dopaminergic neurons in the SNc in RVG9R:pff injected rats, at 6 months post injection (Fig. 4a, b, Supplementary Fig. 4a, b). These pathologies were likely to have resulted from endogenous rodent α-synuclein aggregation, as no human α-synuclein was detected in the host brain at 6 months after the one-off intravenous administration (Supplementary Fig. 4c), consistent with the injected pff seeding host pathology. There was also a progressive decline in noradrenergic cell density in the locus ceruleus (LC), cholinergic neurodegeneration in the dorsal motor nucleus of the vagus nerve (DMN-X), as well as in the hypoglossal nerve (DMN-XII) (Fig. 4d–f). These inclusion bodies in the DMN-X were PK-resistant, LB-like, amorphous precipitates that were reactive to thioflavin-S (Supplementary Fig. 5a). Such inclusions were also ubiquitylated and phosphorylated at the serine-129 residue of α-synuclein (Supplementary Fig. 5b).

### RVG9R:pff treatment generates site-specific and time-dependent α-synucleinopathy

**Fig. 5:**
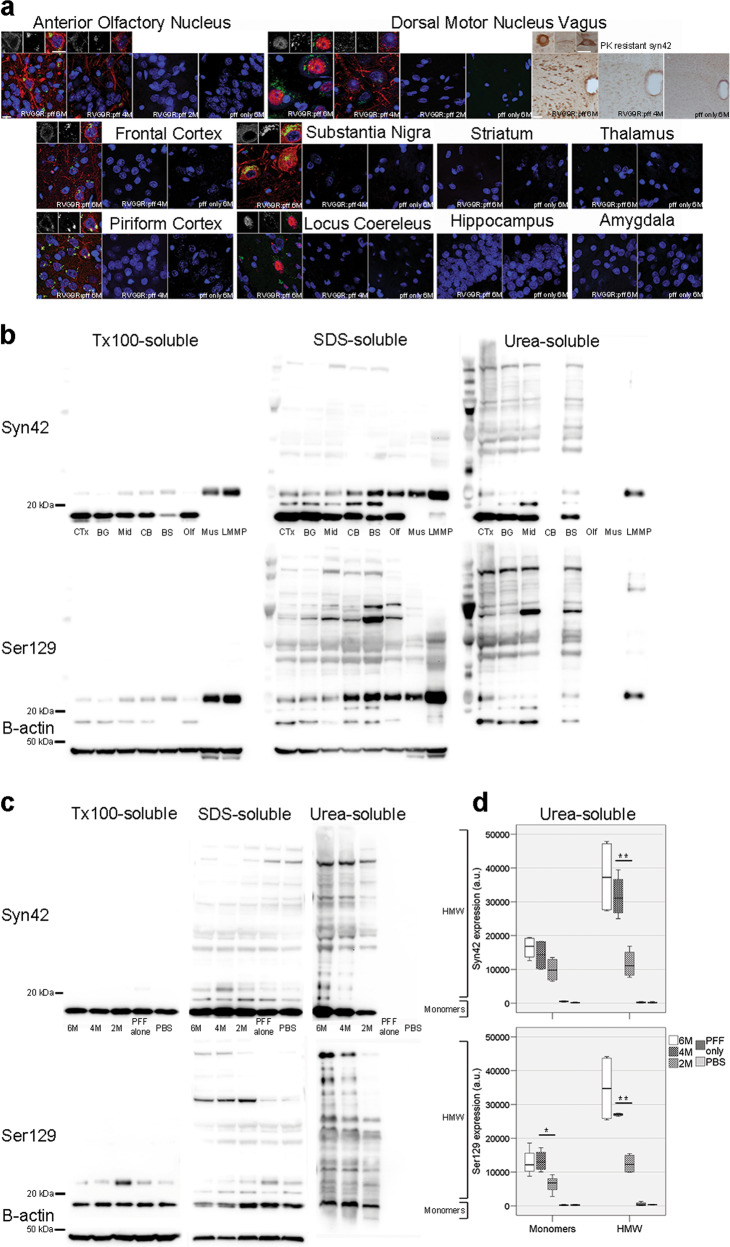
Site-specific and time-dependent α-synuclein pathology after a single systemic injection. **a** Representative figures showing the degree of α-synuclein phosphorylation (Ser129, red) and oligomerization (pFTAA, green) with confocal microscopy in various brain regions, as well as the presence of PK-resistant inclusions in bright-field microscopy in DMN-X. Lewy neurite-like structures, as well as LB-like structures with a dense core of phosphorylated α-synuclein surrounded by pFTAA^+^ oligomers, was detectable in the SNc and DMN-X. *n* = 6–12 per group per time point. **b** Representative blots showing the development of total (syn42) and phosphorylated (Ser129) α-synuclein aggregation after sequential protein extraction, comparing between various regions of the CNS and ENS. At 6 months post injection, total and phosphorylated α-synuclein was detectable in the urea-soluble fraction in all brain regions except for the cerebellum (CB) and OB, as well as in the duodenal LMMP but not in the mucosa. **c** The aggregation and phosphorylation of α-synuclein in the CTx was also time dependent in the urea-soluble fraction. **d** Semi-quantitative analysis on the level of monomeric and HMW α-synuclein in the urea-soluble fraction, at 6 months post injection. There was a significant interaction effect between treatment and α-synuclein conformation (*F*_8,58_ = 5.748, *p* < 0.001). Post hoc analysis revealed no significant difference in the expression of Syn42 α-synuclein monomers between 2 and 6 months post injection, although there was a significant increase in HMW total α-synuclein aggregation between 2 and 4 months post injection. On the contrary, the expression of Ser129 phosphorylated α-synuclein was significantly elevated between 2 and 4 months post injection, both in terms of monomeric (*p* = 0.031), and HMW α-synuclein (*p* < 0.001). Abbreviations include cortex (CTx), basal ganglia (BG), midbrain (Mid), brain stem (BS), and duodenal mucosa (Mus). *n* = 4 per group per time point. Nuclei were counterstained with Hoechst (blue). Scale bars, 30 µm for confocal microscopy and 60 µm for bright-field microscopy

These data provided justification for further analysis, which revealed a differential pattern of α-synuclein pathology across different brain regions. For instance, LB-like, phosphorylated α-synuclein inclusions were observed in the LC and DMN-X, with pFTAA-positive oligomers found surrounding the inclusion bodies (Fig. 5a). The presence of both voluminous and threadlike Lewy pathologies, similar to those described in the dorsal medulla of idiopathic PD patients [22], could be detected in the DMN-X at 6 months after RVG9R:pff injection (Fig. 5a). The presence of α-synuclein pathology could be observed in other brain regions that have previously been implicated in early-stage PD, such as the anterior olfactory nucleus and the SNc. The selective nature of the pathology observed was also evidenced by the absence of pathology at other sites, such as the thalamus and hippocampus, at 6 months after RVG9R:pff injection (Fig. 5a).

Finally, the extent of α-synuclein aggregation was assessed using western immunoblotting. Triton-, SDS-, and urea-soluble α-synuclein were sequentially extracted from different regions of the CNS and ENS. At 6 months after RVG9R:pff injection, Triton-soluble fractions displayed both total and phosphorylated, monomeric α-synuclein in all brain regions and dimeric α-synuclein in the duodenum (Fig. 5b). The presence of high molecular weight (HMW) α-synuclein in the SDS-soluble fraction, with the absence of α-synuclein in the cerebellum, OB, and in the duodenal mucosa in the urea-soluble fraction, indicates that there is a region-specific susceptibility to intravascularly delivered pathogenic pff (Fig. 5b). Using the cortex as an example, aggregation of α-synuclein in RVG9R:pff rats was also found to be a time-dependent process, as both the size and intensity of HMW α-synuclein was progressively increased in the urea-soluble fraction in animals receiving RVG9R:pff (Fig. 5c, d). The presence of phosphorylated α-synuclein in the Triton- and SDS-soluble fractions of PBS-injected animals was likely associated with the membrane fixation process used to enhance the sensitivity of detecting HMW α-synuclein [23].

## Discussion

There is increasing evidence demonstrating that misfolded α-synuclein can spread from one cell to another, possibly through direct synaptic transfer, or via exosomes, tunnelling nanotubes, and *LAG3*-dependent endocytosis [24–27]. The relevance of such spread in the pathogenesis of idiopathic PD however remains debated [28]. In this study, we have developed a novel method to systemically deliver pathogenic α-synuclein pff, and demonstrated that a single intravascular injection using this approach can induce a region-selective and time-dependent neuronal pathology resembling that is seen in premotor, idiopathic PD. This strategy therefore provides a potential model system to study the earliest stages of disease pathogenesis in PD. This not only will improve our understanding of the mechanism and functional relevance of α-synuclein pathogenic spread, but will also be beneficial for the evaluation of novel disease-modifying therapeutics. Moreover, therapeutic proteins could also be delivered using this same carrier system, as we have described here using RVG9R:ChABC and previously with RVG9R:p137 in neurotoxic models of PD [14].

While there are many symptomatic treatments for the dopaminergic aspects of PD, no disease-modifying therapies have yet been successfully translated to the clinic. This is in part due to the lack of animal models recapitulating the earliest stages of this disease. Most neurotoxin-based models are acute in nature without generating α-synucleinopathy. Similarly, transgenic models only represent a small proportion of the familial PD cases, and even in these models, α-synuclein pathology is often not sufficient to induce neurodegeneration at sites relevant to PD [29]. Animal models generated by direct pff inoculation faithfully reproduce many of the pathological features that are seen in the post-mortem brains from idiopathic PD cases [6–9]. Nevertheless, direct intracerebral pff injection never induces pathology outside the brain, at odds with the clinical picture of idiopathic PD in which α-synuclein pathology is abundant in the ENS and may precede that found centrally [30]. Although a recent study has demonstrated that an enteric inoculation of pff generates pathological features in the GI system that propagates into the CNS [31], such findings have not been universally reported [32–34]. Thus, whether ENS abnormalities precede and relate to the onset of motor problems in idiopathic PD is still a matter of debate [35]. Nevertheless, a transvascular delivery of pffs that critically generates pathology only in selective, PD-related CNS and ENS sites, renders our RVG9R:pff model clinically relevant, given its ability to recapitulate the α-synuclein pathology seen in many patients with early-stage PD.

The molecular mechanism causing such differential susceptibility remains elusive. As the propagation of α-synucleinopathy is dependent on the level of α-synuclein of the recipient cells [6], it has been hypothesized that selective neuronal vulnerability may relate to the differential expressions of endogenous α-synuclein. Indeed, glutamatergic neurons in the hippocampus express higher endogenous α-synuclein levels and are more susceptible to PFF toxicity than GABAergic neurons [36]. Similarly, a subset of *Math2*^*+*^ glutamatergic neurons in the hippocampus are more susceptible than *Prox1*-containing glutamatergic neurons in the dentate gyrus, which is again related to the differential expression of endogenous α-synuclein [37]. However, this cannot be the whole story, as many brain areas have similar levels of endogenous α-synuclein yet exhibit selective PD pathology [38].

There are some limitations despite the obvious utility of the RVG9R:pff model. The modest level of nigral dopaminergic cell loss (15–20%) at 6 months post injection was inadequate to generate motor impairments. This likely also explains our MRI findings of no clear brain atrophy following adjustment for multiple comparisons. There were, however, some regional volumetric changes, which correlated with olfactory dysfunction, albeit at an exploratory threshold. For example, the negative correlation between cortical volume and olfactory performance in the RVG9R:pff-exposed rats is in good agreement with similar relationships in non-demented older adults with olfactory impairment [39, 40] and in PD patients with no co-morbid dementia [41, 42], although such changes are not found in all studies [43]. The positive correlations with changes in the pontine nuclei and BNST are slightly harder to interpret, since these regions do not have obvious involvement in olfactory processing. Nevertheless, such changes could be related to neuropathological changes, such as neuronal swelling and gliosis in these regions, associated with systemic RVG9R:pff administration. However, it should be noted that these correlations were exploratory and not corrected for multiple comparisons, and so should be viewed cautiously. Further studies will be required to confirm these relationships and elucidate the cellular correlates driving them. Overall though, our MR imaging endophenotype is consistent with the general lack of structural brain pathology detected by MRI in de novo PD patients [44]. Our data are also consistent with other reports that have injected pff directly into the brain, where the neuropathology and relationship to behavioural dysfunction may become clearer with longer follow-ups coupled to longitudinal in vivo MR imaging.

We did however detect a significant cholinergic neurodegeneration in the DMN-XII at 6 months in the RVG9R:pff rats. Although the presence of ubiquitin-positive degenerating neurites in the DMN-XII has been previously reported in PD patients [45], cholinergic neurodegeneration has not been established in this area. This could be attributed to the intrinsic biological difference between rodent and human cholinergic neurons in the DMN-XII, contributing to their differential susceptibility to α-synuclein pathology. However, a trend of cholinergic neurodegeneration (*p* = 0.073) in the DMN-XII has recently been reported in patients dying with incidental LB disease, but intriguingly not in idiopathic PD patients [22]. Prominent α-synuclein pathology could also be observed in the cortical regions at 6 month after RVG9R:pff injection. It is possible that transvascular delivery of α-synuclein may lead onto cognitive deficits, which define prodromal PD dementia [46], although we did not thoroughly explore this in our study. Furthermore, we used human pff as opposed to rodent pffs, which may be better to generate pathology, as homologous seeding is more efficient and pathogenic than cross-seeding [7].

Another limitation of the current study is that our analysis in the periphery was restricted to describing the behavioural and cellular deficits in the GI tract in the RVG9R:pff animals, and there may be additional pathological features that we did not examine (e.g. heart) [47, 48]. Similarly, our histological examination of the CNS was restricted to the brain regions that have been well established to have pathology in early PD. This was also driven by our MRI analysis. Nonetheless, the datasets presented herein converge to suggest that we have a phenotype at 6 months of age consistent with early-stage PD.

In addition to developing this highly relevant new model of premotor PD, we have also shown that the RVG9R carrier is a versatile system for in vivo protein transduction to complement its previously defined uses in the transvascular delivery of small interfering RNA [13], non-coding RNA [14], and liposome-encapsulated nucleotides [49]. As we have shown here, cargo complexing was simple and achievable within 30 min, in contrast to alternative approaches that require laborious procedures and extensive preparations to create chimeric, CPP-cargo fusion constructs. We have also ascertained that the same delivery system can be adapted to deliver a range of protein cargos including GFP, heterodimeric ChABC, as well as α-synuclein fibrils. In principle the same strategy could also be used to model other neurodegenerative proteinopathies [50].

In summary we have developed a new animal model in which the transvascular delivery of RVG9R:pff mirrors the earliest pathology and behavioural deficits of premotor PD. This opens up new ways to study PD pathogenesis and its treatment, and critically disease-modifying therapies that are likely to have their greatest impact at the onset of the disease process.

## Supplementary information

Supplementary material
