# Supplementary material for: Systemic α-synuclein injection triggers selective neuronal pathology as seen in patients with Parkinson’s disease
Source: Mol Psychiatry. 2019 Nov 22;26(2):556–67. doi: 10.1038/s41380-019-0608-9 (PMC7850975; doi:10.1038/s41380-019-0608-9)
Supplement: Supplementary file 1 — Supplementary material [file 41380_2019_608_MOESM1_ESM.docx]

**Supplementary Material**

**Systemic α-synuclein injection triggers selective neuronal pathology as seen in patients with Parkinson’s disease**

Wei-Li Kuan, Katherine Stott, Xiaoling He, Tobias C. Wood, Sujeong Yang, Jessica C.F. Kwok, Katie Hall, Yanyan Zhao, Ole Tietz, Franklin I. Aigbirhio, Anthony C. Vernon, and Roger A. Barker

**Summary**

The supplementary material includes Detailed Materials and Methods, and 5 supplementary figures

**Detailed Materials and Methods**

**Ethics.** Human fetal tissue was collected from routine termination of pregnancies at the Cambridge University Hospitals (Addenbrooke’s) and experiments performed at the John van Geest Centre for Brain Repair (University of Cambridge). All human tissue was collected under full ethical approval in accordance with the United Kingdom's Department of Health guidelines and local ethical approval (NRES Committee East of England, 96/085). Informed consents were obtained from all donors. All animal experiments were done in accordance with a project licence held under the Animals (Scientific Procedures) Act 1986, Amendment Regulations 2012, following ethical review by the University of Cambridge animal welfare and ethical review body.

**Peptides.** Peptide RVG9R (YTIWMPENPRPGTPCDIFTNSRGKRASNGGGGRRRR RRRRR) comprises of a short domain originating from the rabies virus glycoprotein with nine C-terminally conjugated ᴅ-arginines [13]. At least five independent batches of peptides were used in the experiments described here and were synthesized by ProImmune Ltd (UK) and ProteoGenix SAS (France). Peptides were supplied as a lyophilized powder. For use experimentally, it was suspended in water at a concentration of 20mg/ml and stored in aliquots at -80°C.

**Proteins.** Full length, wild-type human recombinant α-synuclein and full length, human α-synuclein uniformly labelled with ^15^N for NMR spectroscopy, were both purchased from rPeptide. Full length, active recombinant GFP protein was originally derived from *Aequoreavictoria* and purchased from Abcam. Active ChABC enzyme was purified from *Proteus vulgaris* and purchased from Sigma-Aldrich.

**Cell cultures.** For primary rat or human fetal cultures, cortices from the E18 rat fetus or 61-70 day old human fetus were dissected under sterile conditions, mechanically triturated into a single cell suspension, and seeded onto poly-L-lysine-coated glass coverslips pretreated with 50μg/mL laminin (Sigma-Aldrich). Cells were maintained in DMEM supplemented with 10% FBS and 2% B-27 (Gibco), at 37°C in 5% CO­_2_. After 7 days in culture, peptide/protein complexes were added to the culture medium for another 24hr, before cells were fixed for immunocytochemical analysis. Protein cargos were incubated at a density of 1µg of protein per 50000 cells. Cells were fixed using 4% PFA for 20min in room temperature.

**Analytical ultracentrifugation.** Sedimentation velocity experiments were conducted with an Optima XL-I (Beckman Coulter) centrifuge using an An60Ti four-hole rotor. Standard double-sector Epon centrepieces equipped with sapphire windows contained either 400μL of α-synuclein or α-synuclein with RVG9R at a concentration of 30μM. UV absorbance data were acquired in the continuous mode at time intervals of 560s and a rotor speed of 40,000rpm, at a temperature of 20°C with the systematic noise subtracted. The density and viscosity of the buffer (PBS) and the partial specific volume of the protein(s) were calculated using Sednterp [51]. Multi-component sedimentation coefficient distributions were obtained from 128 scans by direct boundary modelling of the Lamm equation using SEDFIT v.14.1 [52].

**Protein NMR Spectroscopy.** NMR measurements were made on ^15^N-labelled α-synuclein (starting concentration 91μM) in 10% D_2_O, 20mM sodium phosphate (pH 6.0). Protein concentrations were estimated from the measured A_280_ using the calculated extinction coefficients. Experiments were performed at 293*K* on a Bruker AVANCE 600MHz spectrometer. Data were processed using the AZARA suite of programs (v. 2.8, © 1993-2017; Wayne Boucher and Department of Biochemistry, University of Cambridge, unpublished). Backbone assignments were made with reference to the assignments of Bax *et al*. using CcpNmr Analysis v. 2.4 [18,53]. Chemical-shift differences were calculated using Δδ=[(Δδ^H^)^2^ + (0.15 ×Δδ^N^)^2^]^1/2^.

**Determination of dissociation constant (*K*_d_).** *K*_d_ values were calculated from the fitting of the NMR data using CcpNmr Analysis v. 2.4 to Δδ = *A*{*B*+*x*–√((*B*+*x*)^2^–4*x*)}, where Δδ is the chemical-shift difference (see above), *A*=Δδ_∞_/2, *B*=1+*K*_d_/[α-synuclein]_total_ and *x*=[RVG9R]_total_/[α-synuclein]_total_. The reported estimated *K*_d_ was obtained by averaging the fitted values over residues 120-140 at the C-terminus. An estimate of *k*_off_ was obtained from the *K*_d_ (80μM) using the rate of approach to equilibrium [54], *k*_obs_ ≈ *k*_off_+(*k*_on_×[ligand]), given *k*_on_ = *k*_off_/*K*_d_, and the concentration for the NMR experiments was 91μM, *k*_obs_ ≈ *k*_off_ + (*k*_on_ × [ligand]) ≈ *k*_off_ + (*k*_off_/80×10^-6^) × (91×10^-6^)= 2.1 × *k*_off_. Moreover, equilibrium was achieved quickly, i.e. no change to the NMR spectrum was observed in the instrument within 2min of adding RVG9R for each titration point. This places a conservative lower limit of 2.1 × *k*_off_ of 1/120 seconds = 0.008s^-1^, i.e. *k*_off_> 0.004s^-1^. Put another way, the longest possible value of the dissociative half-life of the peptide/protein complex is *ln*2/*k*_off_ i.e. *c*. 170s.

**Preparation of α-synuclein pff.** α-synuclein pff were prepared using the protocol published previously [55]. In short α-synuclein monomers (rPeptide) were resuspended in 10mM Tris-HCl, 50mM NaCl, pH 7.6 at a concentration of 5μg/μl. They were then placed on a 37°C thermomixer, agitated for 7 days at 1,000 r.p.m, and then transferred to a -80°C freezer. Immediately before use the pffs were thawed and diluted to 1μg/μl using PBS, and sonicated with 60 pulses at 10% power for 30s with a Soniprep 150 Plus ultrasonic disintegrator (MSE).

**Transmission electron microscopy (TEM).** α-Synuclein pff samples, before and after sonication, were adsorbed onto glow discharged (GlowCube, Quorum Technologie Ltd, Laughton, UK.) 400 mesh, copper, carbon film grids (EM Resolutions, Saffron Walden, UK) for 30s, rinsed twice on DIW and negatively stained with 1.5% aqueous uranyl acetate for 30s. They were blotted on filter paper and viewed in a FEI Tecnai G2 (FEI Ltd, Oregon, USA operated at 20kV). Images were recorded with an AMT XR60B camera running Deben software (AMT, Woburn, USA).

**Behavioral assessments.** Solid gastrointestinal (GI) retention was examined using resin beads as described previously [56]. In short, resin beads (diameter 0.25-0.8mm, Diaion HP-20, Sigma-Aldrich) were colored with 1% Evans blue dye (Sigma-Aldrich) before use to enhance visibility in the GI tract. Animals were fasted overnight and given 300µL of 3% resin beads in 10% sucrose orally. After 4hrs the whole small intestine was removed. GI transit (%) was measured as the distance travelled most caudally by the beads expressed as a length between the pylorus and the ileocecal valve. For the buried food test, animals were familiarized with the food stimuli (Kellogg's coco pops) and fasted overnight. A food pellet was then buried in the middle of the home cage and the latency to retrieve the pellet was measured. If the subject failed to find the buried food after 15min, the test was stopped with 900s recorded as its latency score [57]. An odor cross-habituation test was performed as described previously [58]. In short, the odors used (limonene and pentanol, Sigma-Aldrich) were diluted 1 × 10^-3^ into mineral oil and 20μL of odor was applied to 3mm filter paper (Whatman). Odors were presented on three successive occasions for 2min, separated by 30s intervals. The presentation of odors was counterbalanced in testing groups and the raw values were normalized to the maximum investigatory duration during the three trials for each odor. The discrimination index was calculated by subtracting the normalized duration of the previous trial odor investigation (third trial) from that of the following first trial presentation. Gross sensorimotor function of the animal’s forelimb was studied using the adjusted stepping test [59]. Briefly the experimenter held both hindlimbs and one forelimb of the animal and moved the rat sideways across a platform over a metre. The unrestrained forelimb was in contact with the platform and the number of adjustment steps performed by the rats counted in the forehand direction. Gross motor coordination was studied using the ladder climbing test. In brief, the rats were placed on top of a wired shelving ladder (1.5 x 0.3m, 60% angle from the ground) and were subjected to two trials. Both the habituation and descent latencies were recorded. Repetitive digging behaviour was investigated using the marble burying test. Clean cages were filled with 4.5cm corncob bedding, followed by gently overlaying of 20 black glass marbles (15mm diameter) equidistant in a 4×5 arrangement. Animals were placed individually in the cage and after a 30min exploration period, the number of totally unburied marbles and mostly buried marbles (>50% marble covered by bedding material), were recorded. Individual animals were subject to all the behavioural assessments described, which were performed during the dark phase of a 12 hr light/dark cycle.

***Ex vivo* structural MRI preparation.** Animals were culled at 6 months of age by cardiac perfusion (0.9% saline followed by 4% paraformaldehyde) under terminal anaesthesia (sodium pentobarbital, 60 mg/kg i.p). Brain tissues were harvested and prepared for *ex vivo* MRI as described elsewhere [60]. In brief, perfusion-fixed brain tissues were kept intact in the cranium and post-fixed for 24 hours in 4% PFA. Samples were then placed in 0.01M phosphate buffer containing 0.05% (w/v) sodium azide to allow tissue re-hydration prior to MRI. Samples were then shipped to Kings College in London and stored at 4°C in this solution for 4 weeks prior to MRI.

**MR image acquisition.** A 7T horizontal small bore magnet (Agilent Technologies Inc. Santa Clara, USA) and a quadrature volume radiofrequency coil (39 mm internal diameter, Rapid Biomedical GmbH, GER) were used for all MRI acquisitions. Fixed brain samples were placed securely, one at a time, in a custom-made MR-compatible holder and immersed in proton-free susceptibility matching fluid (FluorinertTM FC-70; Sigma-Aldrich). Samples were scanned in a random order, with the operator blinded to treatment group by numerical coding of samples. Scanning was interspersed with phantoms to ensure consistent operation of the scanner. T2-weighted 3D Fast Spin Echo (FSE) MR images were acquired with the following parameters: TE/TR=60/2000, echo train length=8, matrix size=192x128x192 and field of view (FOV)=28.8x19.2x28.8 mm, yielding isotropic voxels of 150 µm^3^. Total scan-time per brain was 1hr 44min.

**MR image processing.** MR images were visually inspected in native space for artefacts, with no images excluded on this basis. Raw MR Images were converted from the manufacturer’s proprietary format to the NIFTI format and processed using a combination of FSL [61], ANTs [62] and in-house C++ software utilizing the ITK library, available from *https://github.com/spinicist/QUIT*. The processing pipeline consisted of several steps, as described elsewhere [63,64]. The following steps were performed on the MR images in their native space. A Tukey filter was applied to the FSE MR images in k-space to remove high frequency noise followed by correction for intensity inhomogeneity using the N4 algorithm [65]. A template image was then constructed from the 3D FSE images of all subjects in the study, using the *antsMultivariateTemplateConstruction2.sh* script with cross-correlation metric and SyN transform [66]. This template was then registered to an atlas image of the adult Sprague-Dawley rat brain in Waxholm space, again using a cross-correlation metric and SyN transform [67]. The FSE images for all study subjects were non-linearly registered to the study template using the *antsRegistrationSyN.sh* script. Logarithmic Jacobian determinants were calculated from the inverse warp fields in standard space to estimate apparent volume change and smoothed with a Gaussian filter at a full-width half-maximum (FHWM) of 200 µm [68]. A brain parenchyma mask was then created from the atlas labels by excluding cerebrospinal fluid regions [67]. The inverse transforms from the atlas to the study template, and from the study template to each subject, were applied to calculate the brain and atlas based ROI volumes for each subject [63,64].

**MR image statistics.** Group-level differences in volume (mm^3^) were assessed using a combination of atlas-based segmentation (ABS) and voxel-wise tensor-based morphometry (TBM) [69]. For ABS, we utilised a publicly available high resolution MRI atlas of the SD rat brain pre-parcellated into 80 ROIs [66]. After image registration and careful checking of atlas label alignment to each individual subject’s MR images, we automatically extracted values for volume for 79 ROIs. Total brain volume was calculated from the summation of each individual atlas ROI volumes [69]. We first created and analysed summary “grey” and ”white” matter ROIs, comprised of all atlas ROIs for each tissue component, before then comparing the individual atlas ROIs. For all comparisons we used relative volumes (expressed as a percentage of total brain volume), since total brain volume accounts for the majority of inter-animal variation in the volume of individual brain structures [70,71]. Group-level differences between control and RVG9R:pff-exposed animals were assessed using multiple 2-tailed t-tests (unequal variance assumed) with α=0.05. The resulting *p*-values were then corrected for multiple comparisons (to account for Type I errors across the 2 summary and 79 individual ROIs using the False Discovery Rate (FDR) procedure, with the threshold set at 5% (*q*<0.05). Effect sizes were calculated using Glass’ delta. Exploratory correlations between total and regional brain volumes and performance in the buried food test were modelled using bivariate Pearson’s Product moment correlations. Since this was an exploratory analysis, the *p*-values derived from these correlations were not corrected for multiple comparisons. For TBM, voxel-wise analysis of group-level differences were carried out on the Jacobian determinant images, using permutation tests and Threshold-Free Cluster Enhancement (TFCE) with FSL *randomize,* corrected for multiple comparisons using the family-wise error rate (FWE *p*<0.05) [72]. Total brain volume estimates were included as a regressor of no interest in the design matrix.

**Synthesis of pFTAA.** The synthesis of pFTAA was carried out at the Molecular Imaging Chemistry Laboratory, University of Cambridge and followed the synthetic strategy described previously with slight modifications [21]. The synthetic strategy employed ethyl-ester protected building blocks. The commercially available starting material, Ethyl 3-thiopheneacetate, was brominated to yield Ethyl 2-(2-bromothiophen-3-yl)acetate, which in turn was Suzuki cross-coupled to commercially available 2,5-bis-thiopheneboronic acid pinacol ester to yield the ethyl-ester protected trimer, which was subsequently brominated. The ethyl-ester protected pentamer was synthesized by Suzuki cross-coupling of the brominated trimer and commercially available 5-(ethoxycarbonyl)thiophene-2-yl)boronic acid; the compound was purified by silica column chromatography and subsequently deprotected using NaOH to yield pFTAA. Relevant spectroscopy data of the synthesized compound is provided below:

^1^H-NMR (300 MHz, CD_3_OD): 3.66 (s, 4H, C*H*_2_); 7.16 (d, J=3.8Hz, 2H, Ar-*H*); 7.29 (s, 2H, Ar-*H*); 7.32 (s, 2H, Ar-*H*); 7.47 (d, J=3.9Hz, 2H, Ar-*H*);

^13^C-NMR (75 MHz, d_6_-DMSO)): 38.4, 123.1, 126.7, 128.0, 130.2, 130.6, 134.9, 135.7, 135.8, 140.1, 141.9, 168.4, 177.4;

LR-MS: 617.2 [ES+]; 615.2 [ES-].

**Immunohistochemistry.** To separate longitudinal muscle myenteric plexus (LMMP) from the underlying mucosal layer containing the submucosal plexus (SP), the duodenum was cut into small segments (2-4cm each) and extensively rinsed to remove GI contents. Segments were then placed on a glass rod, the mesentery was removed, and the longitudinal muscle layer was gently teased away using a cotton swab [73]. After perfusion of the rats, the brains were also removed, post-fixed overnight, and then transferred to 30% sucrose until they sank. Sections were cut at 40µm in either the coronal or sagittal plane, and a 1:6 series of sections was immunostained. After blocking with 0.3% Triton X-100 with 5% serum for 1hr, coverslips or sections were incubated overnight with various primary antibodies including human-specific α-synuclein syn211 (1:1000, Millipore 36-008), α-synuclein LB509 (1:500, Abcam ab27766), phosphorylated α-synuclein Ser129 (1:500, Millipore MABN826), CSPG stub 1B5 (1:80, Amsbio 270431-CS), link protein Crtl1 (1:250, Millipore MABT85), lectin from WFA (1:200, Sigma-Aldrich L1516), GFP (1:500, Abcam ab290), GFAP (1:1000, Dako Z0334), the enteric neuronal marker peripherin (1:500, Millipore ab1530), the catecholaminergic neuronal marker TH (1:1000, Pel-Freez P40101-150), vesicular monoamine transporter-2 VMAT2 (1:1000, Abcam ab70808), cholinergic neuronal marker ChAT (1:250, Millipore AB144P), noradrenergic neuronal marker DBH (1:1000, Genetex GTX101616), and pentameric formyl thiophene acetic acid pFTAA (3µM). Stainings were visualized either by immunofluorescence (Alexa 488, Alexa 568 and Alexa 647, 1:1000, Molecular Probes) or by diaminobenzidine (DAB). For proteinase-K (PK) treatment, free-floating sections were incubated in PBS containing 10µg/ml of PK (Invitrogen) for 30min at 37°C before blocking, and overnight incubation with LB509 antibody [74]. For whole mount LMMP and SP staining, sections were subjected to PK treatment, followed by blocking with 0.3% Triton X-100 with 5% serum/2% BSA/0.08% sodium azide for 5 days. Primary and secondary antibodies were added at room temperature for 24hrs, and mounted on Nunc™ Lab-Tek™ II chamber slides with a silicon gasket due to section thickness. Staining was also done in all experiments without the primary antibody such that we had a negative control in all cases. For thioflavin-S (ThS) staining, sections were first mounted and rehydrated in decreasing concentrations of ethanol. Sections were then incubated for 60min at room temperature with 0.05% ThS, which had been diluted in distilled water and filtered. Sections were then differentiated for 3min in escalating levels of ethanol, treated with 0.2% Sudan black B (Sigma) in 70% ethanol for 30s, and finally washed in PBS.

**Monoamine analysis.** Olfactory bulb (OB) samples were homogenized in 0.2 M perchloric acid, and centrifuged at 1,000*g* at 4°C for 20min. Twenty-five microliters of the supernatant was injected onto the HPLC-ECD system to measure levels of dopamine, noradrenaline, and serotonin. Detection and quantifications were achieved using a Coulochem II detector with an analytical cell and two electrodes in series (E1 -250mV, E2 +250mV). The signal from E2 was integrated using computer software (Chromeleon, Dionex, UK).

**Sequential protein extraction.** Cellular proteins from the brain or peripheral tissues were extracted using a Dounce homogenizer in 1% Triton X100 supplemented with Complete protease inhibitor (Roche). Total protein concentrations were determined for the Tx100- and SDS-soluble fractions using the BCA assay. After centrifugation at 1000*g* at 4°C for 10min, the samples were centrifuged at 120,000*g* using an Optima MAX-XP ultracentrifuge (Beckman Coulter) for 60min at 4°C. The supernatants were collected (Tx-100 soluble fraction) and the precipitates were resuspended in 5% SDS/1% Triton X100 at 120,000*g* for 60min at 4°C. The supernatants were again collected (SDS soluble fraction) while the precipitate was resuspended in 8M urea/5% SDS/1% Tx100 to give the urea-soluble fraction. Protein concentration was determined by the BCA assay (Life Technologies) for the Tx100- and SDS-soluble fractions before proceeding to electrophoresis.

**Western blot.** Cellular proteins from the brain or peripheral tissues were extracted using a proprietary extraction buffer based on Triton X-100 and SDS (Abcam), supplemented with Complete protease inhibitor. Total protein concentration was determined by the BCA assay. A total amount of 20-30µg of protein per lane was loaded onto a 10% pre-cast gel (ThermoFisher) for SDS-PAGE and electroblotted onto 20nm polyvinylidene difluoride membranes (GE). For α-synuclein blotting there was an additional step to fix the membrane in 4% PFA for 60min prior to blocking. Membranes were then blocked in TBS Tween buffer (Pierce) supplemented with 5% dry skimmed milk, and incubated with antibodies against GFP (Abcam ab290), total α-synuclein syn42 (1:2500, BD Biosciences 610787), phosphorylated α-synuclein Ser129 (1:2500, Abcam ab51253), GFAP (1:1000, Dako Z0334), peripherin (1:1000, Millipore ab1530), TH (1:1000, Millipore AB152), or β-actin (1:5000, Santa Cruz sc-47778) at 4°C overnight. The membranes were then rinsed and incubated with horseradish-peroxidase-conjugated secondary antibody (1:5000, Santa Cruz) for 1hr at room temperature. Membranes were developed with the SuperSignal West Pico chemiluminescence method (Life Technologies), and then detected by exposing the membrane in the Bio-Rad UV-imaging suite from 30 to 900s.

**Microscopy, stereological, and statistical analysis.** Imaging was performed using a DM6000 microscope or a TCS SP2 confocal microscope (both from Leica). The total number of dopaminergic neurons in the substantia nigra pars compacta (SNc) and cholinergic neurons in the DMN, as well as the density of noradrenergic neurons in the LC, was estimated by unbiased stereology using a combination of the cavalieri probe and optical fractionator, with the Stereo Investigator 9.10.3 software (MBF Bioscience). For statistical analysis, data normality was verified using either the Kolmogorov-Smirnov (>50 samples) or the Shapiro-Wilk (≤50 samples) tests. The assumption of homogeneity of variance was tested using Levene's Test. Data transformation was performed to obtain normality when required (such as square root transformation). All quantitative data was analyzed using either a multivariate ANOVA or repeated measure ANOVA as stated. *Post-hoc* analysis was performed using either the Dunnett’s or Bonferroni test (two-tailed) when appropriate, with significance set at *p*<0.05. All analyses were performed using SPSS Release 23.0.0. Data are presented either in box plot showing group median and 1.5 interquartile range, or line graph showing group mean and standard error of the mean. No statistical methods were used to predetermine sample sizes, but our sample sizes are similar to those reported in previous publications [6,7,8]. Group samples were not randomized; data collection, but not data analysis, was performed blind to the conditions of the experiments. No collected data was excluded from analysis.

**References for the supplementary material**

1. Laue T, Shah B, Ridgeway T, Pelletier S. (1992). Computer-aided interpretation of analytical sedimentation data for proteins. In: Harding SE et al. eds. *Analytical ultracentrifugation in biochemistry and polymer science*, The Royal Society of Chemistry, Cambridge, UK, 1992, pp 90-125.
2. Schuck P. Size-distribution analysis of macromolecules by sedimentation velocity ultracentrifugation and lamm equation modeling. *Biophys J*. 2000; 78:1606-1619.
3. Vranken WF, Boucher W, Stevens TJ, Fogh RH, Pajon A, Llinas M, et al. The CCPN data model for NMR spectroscopy: development of a software pipeline. *Proteins*. 2005;59: 687-696.
4. Tummino PJ, Copeland RA. Residence time of receptor-ligand complexes and its effect on biological function. *Biochemistry*. 2008;47: 5481-5492.
5. Volpicelli-Daley LA, Luk KC, Lee VM. Addition of exogenous ​α-synuclein preformed fibrils to primary neuronal cultures to seed recruitment of endogenous ​α-synuclein to Lewy body and Lewy neurite–like aggregates. *Nat Protoc*. 2014;9: 2135-2146.
6. Ando K, Takagi K, Tsubone H. Enhanced gastric retention of solid resin beads as a marker for emetic potential of agents in rats. *J Toxicol Sci*. 2012;37: 549-553.
7. Hall K, Yang S, Sauchanka O, Spillantini MG, Anichtchik O. Behavioural deficits in transgenic mice expressing human truncated (1-120 amino acid) alpha-synuclein. *Exp Neurol*. 2015;264: 8-13.
8. Yang S, Kuan WL, Spillantini MG. Progressive tauopathy in P301S tau transgenic mice is associated with a functional deficit of the olfactory system. *Eur. J. Neurosci*. 2016;44: 2396-2403.
9. Kuan WL, Lin R, Tyers P, Barker RA. The importance of A9 dopaminergic neurons in mediating the functional benefits of fetal ventral mesencephalon transplants and levodopa-induced dyskinesias. *Neurobiol Dis.* 2007;25: 594-608.
10. Vernon AC, Crum WR, Lerch JP, Chege W, Natesan S, Modo M, et al. Reduced cortical volume and elevated astrocyte density in rats chronically treated with antipsychotic drugs-linking magnetic resonance imaging findings to cellular pathology. *Biol Psychiatry*. 2014;75: 982-990.
11. Jenkinson M, Beckmann CF, Behrens TE, Woolrich MW, Smith SM. FSL. *Neuroimage*. 2012;62: 782-790.
12. Avants BB, Tustison NJ, Song G, Cook PA, Klein A, Gee JC. A reproducible evaluation of ANTs similarity metric performance in brain image registration. *Neuroimage*. 2011;54: 2033-2044.
13. Richetto JR, Chesters A, Cattaneo MA, Labouesse AMC, Gutierrez TC, Wood TC. et al. Genome-Wide Transcriptional Profiling and Structural Magnetic Resonance Imaging in the Maternal Immune Activation Model of Neurodevelopmental Disorders. *Cereb Cortex*. 2017;27: 3397-3413.
14. Wood TC, Simmons C, Hurley SA, Vernon AC, Torres J, Dell'Acqua F, et al. 2016. Whole-brain ex-vivo quantitative MRI of the cuprizone mouse model. *PeerJ*. 2016;4: e2632.
15. Tustison NJ, Avants BB, Cook PA, Zheng Y, Egan A, Yushkevich PA, et al. N4ITK: improved N3 bias correction. *IEEE Trans Med Imaging*. 2010;29: 1310-1320.
16. Avants BB, Yushkevich P, Pluta J, Minkoff D, Korczykowski M, Detre J, et al. The optimal template effect in hippocampus studies of diseased populations. *Neuroimage*. 2010;49: 2457-2466.
17. Papp EA, Leergaard TB, Calabrese E, Johnson GA, Bjaalie JG. Waxholm Space atlas of the Sprague Dawley rat brain. *Neuroimage*. 2014;97: 374-386.
18. Cox RW. AFNI: software for analysis and visualization of functional magnetic resonance neuroimages. *Comput Biomed Res*. 1996;29: 162-173.
19. Crum WR, Sawiak SJ, Chege W, Cooper JD, Williams SCR, Vernon AC. Evolution of structural abnormalities in the rat brain following in utero exposure to maternal immune activation: A longitudinal in vivo MRI study. *Brain Behav Immun*. 2017;63: 50-59.
20. Lerch JP, Gazdzinski L, Germann J, Sled JG, Henkelman RM, Nieman BJ. Wanted dead or alive? The tradeoff between in-vivo versus ex-vivo MR brain imaging in the mouse. *Front Neuroinform*. 2012;6: 6.
21. Ma D, Holmes HE, Cardoso MJ, Modat M, Harrison IF, Powell NM, et al. Study the Longitudinal in vivo and Cross-Sectional ex vivo Brain Volume Difference for Disease Progression and Treatment Effect on Mouse Model of Tauopathy Using Automated MRI Structural Parcellation. *Front Neurosci*. 2019;13: 11
22. Smith SM, Nichols TE. Threshold-free cluster enhancement: addressing problems of smoothing, threshold dependence and localisation in cluster inference. *Neuroimage*. 2009;44: 83-98.
23. Smith TH, Ngwainmbi J, Grider JR, Dewey WL, Akbarali H. An in-vitro preparation of isolated enteric neurons and glia from the myenteric plexus of the adult mouse. *J Vis Exp*. 2013;78.
24. Fernagut PO, Hutson CB, Fleming SM, Tetreaut NA, Salcedo J, Masliah E, et al. Behavioral and histopathological consequences of paraquat intoxication in mice: effects of alpha-synuclein over-expression. Synapse. 2007;61: 991-1001.

**
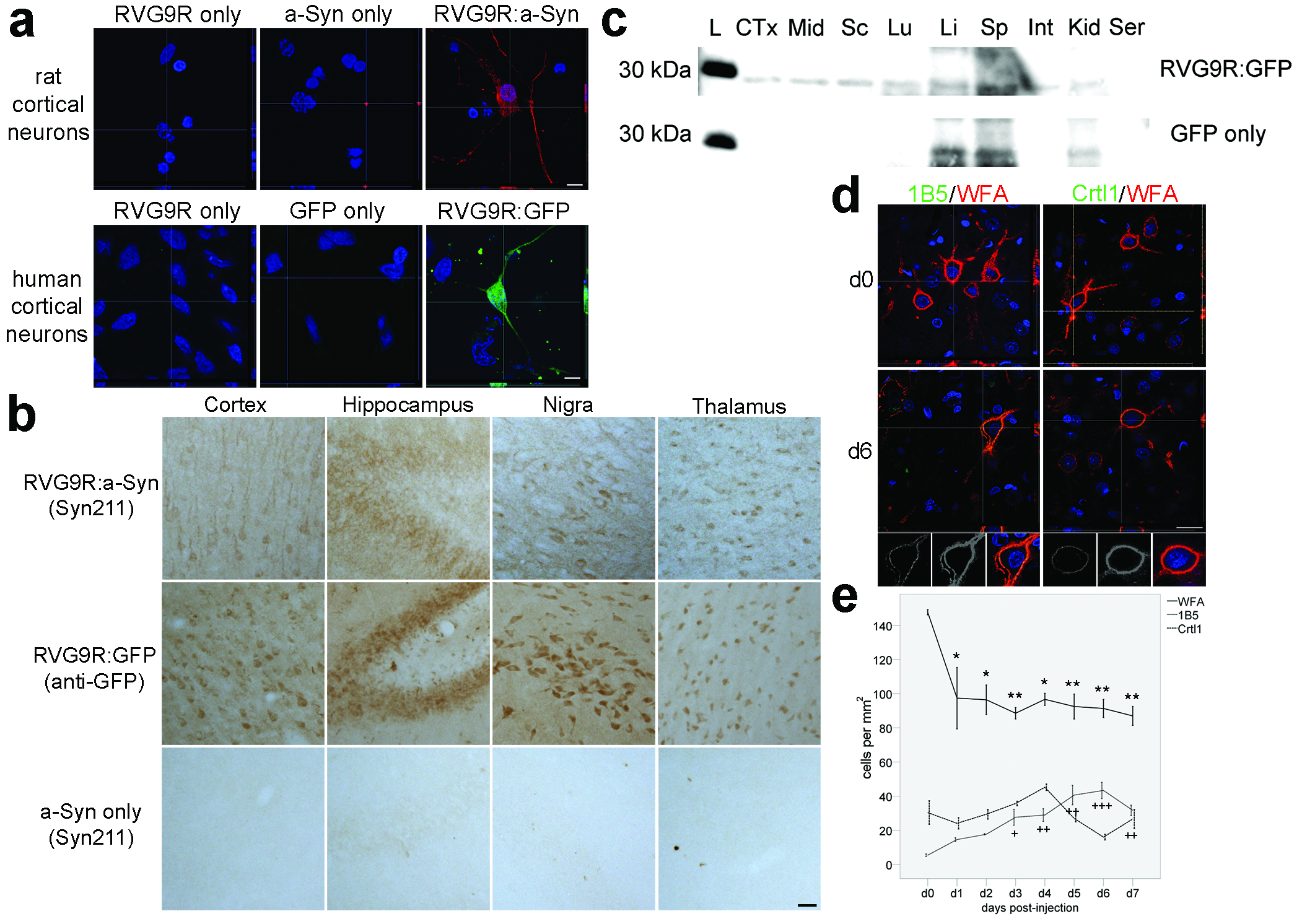
**

**Supplementary Figure 1: RVG9R-mediated protein transduction *in vitro and in vivo*.** (A) Human α-synuclein (syn211, red) was detected in the rat fetal cortical (CTx) cells at 24hrs after incubation when complexed with RVG9R. Likewise, recombinant GFP (endogenous fluorescence, green) was detected in human fetal cortical cells when complexed with RVG9R. RVG9R-delivered GFP emitted green fluorescence, indicative of a proper folding of the cargo protein. *n*=6 per group. (B) Expression of human α-synuclein and GFP in various brain regions at 24hrs after intravenous injection. *n*=3 per group. (C) Representative blots simultaneously exposed to demonstrate the biodistribution of RVG9R-mediated GFP expression at 24hrs after intravenous injection. Abbreviations for protein molecular weight marker (L), cortex (CTx), midbrain (Mid), spinal cord (Sc), lungs (Lu), liver (Li), spleen (Sp), duodenum (Int), kidney (Kid), serum (Ser). *n*=3 per group. (D) Immediately after intravenous RVG9R:ChABC administration (day 0), the PNNs (WFA, red) in the somatosensory cortex were intact with little 1B5 or Crtl1 (both in green) staining. The levels of digested CSPG were substantially increased at day 6 post-injection. (E) Time-course analysis revealed a progressive reduction in WFA (*F*_7,16_=5.835, *p*=0.002), post-hoc analysis between d0 versus d1 (*p*=0.014), d2 (*p*=0.012), d3 (*p*=0.003), d4 (*p*=0.012), d5 (*p*=0.006), d6 (*p*=0.005), d7 (*p*=0.002). There was also concomitant elevation in 1B5 (*F*_7,16_=13.201 *p*<0.001), post-hoc analysis between d0 and d3 (*p*=0.013), d4 (*p*=0.007), d5 (*p*=0.001), d6 (*p*<0.001), d 7 (*p*=0.002). Data represents mean±SEM, * *p*<0.05, ** *p*<0.01 (WFA) and ^+^*p*<0.05, ^++^*p*<0.01, ^+++^*p*<0.001 (1B5) compared with d0. Data was analyzed using a multivariate ANOVA, *n*=3 animals per group per time point. For each animal the number of cells was randomly counted and averaged from 30 sites in the somatosensory cortex. Nuclei were counterstained with Hoechst (blue). Scale bars, 30µm (90µm in B).

**
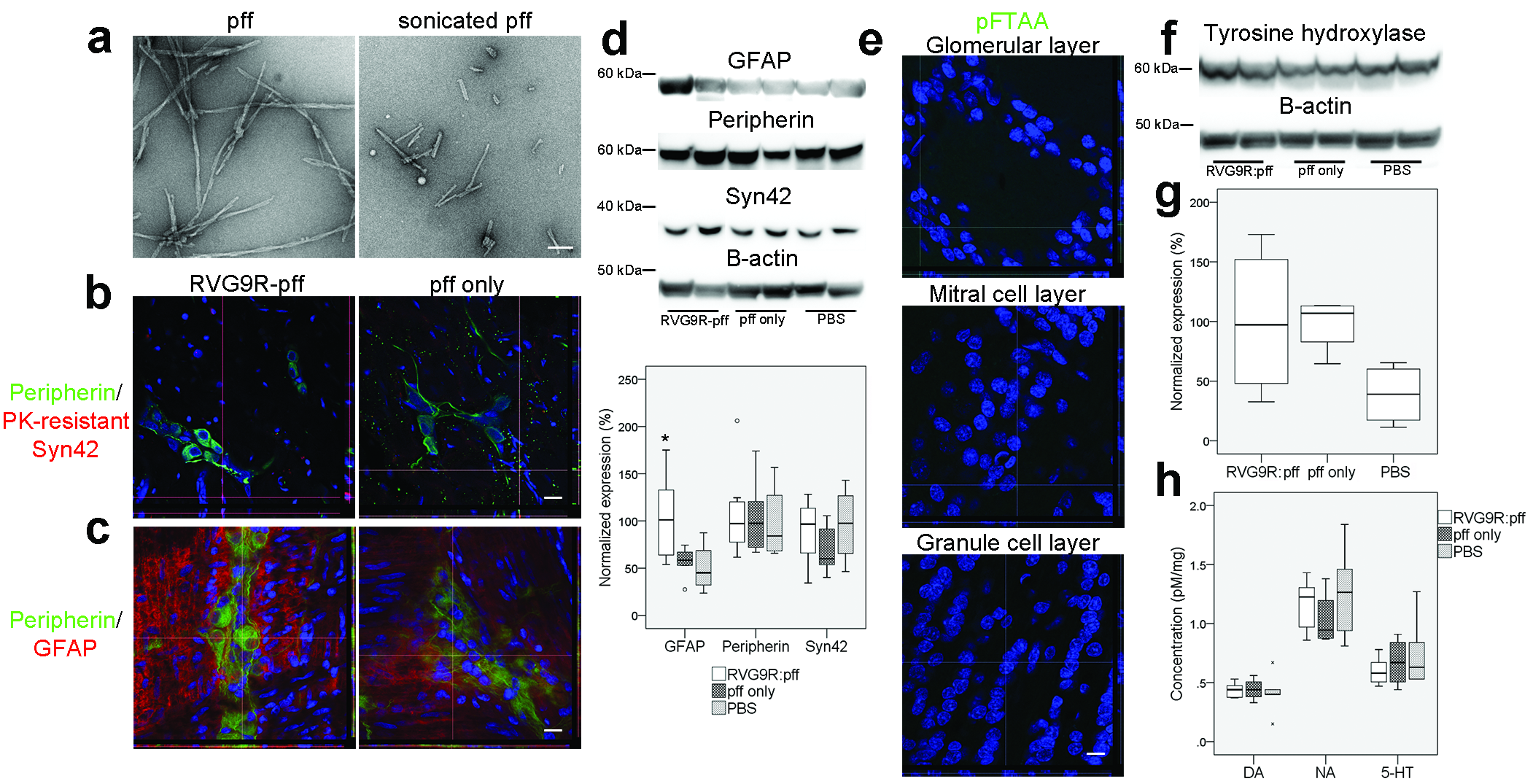
**

**Supplementary Figure 2: The effects of RVG9R:pff treatment on the GI tract and the olfactory system.** (a) Representative TEM images showing the ultrastructure of pff. (b) Unlike those observed in the myenteric plexus, PK-resistant α-synuclein inclusions (red) could not be found in the submucosal plexus (peripherin, green) of the duodenum at 6 months post-lesion. (c) The level of GFAP (red) surrounding the myenteric plexus (green) was upregulated in the RVG9R:pff rat duodenum. (d) Representative blot and (e) the level of GFAP, peripherin, and total α-synuclein (syn42) were semi-quantitatively analyzed in the duodenal LMMP, confirming the presence of astrogliosis in rats receiving RVG9R:pff treatment (*F*_2,17_=5.293, *p*=0.012). Post-hoc analysis between the RVG9R:pff *vs* pff only (*p*=0.028) and PBS (*p*=0.04) groups at 6 months post-lesion. (e) No pFTAA signal (green) was found in the OB. (f) Representative blot and, (g) TH expression was semi-quantitatively analyzed. (h) HPLC analysis revealed no significant changes in the concentration of dopamine (DA), noradrenaline (NA), and serotonin (5-HT), in the OB. **p*<0.05, compared with PBS group at the same time point. Data was analyzed using multivariate ANOVA. *n*=4-8 per group in (d, f-h). Nuclei were counterstained with Hoechst (blue). Scale bars, 30µm (100nm in a).

**
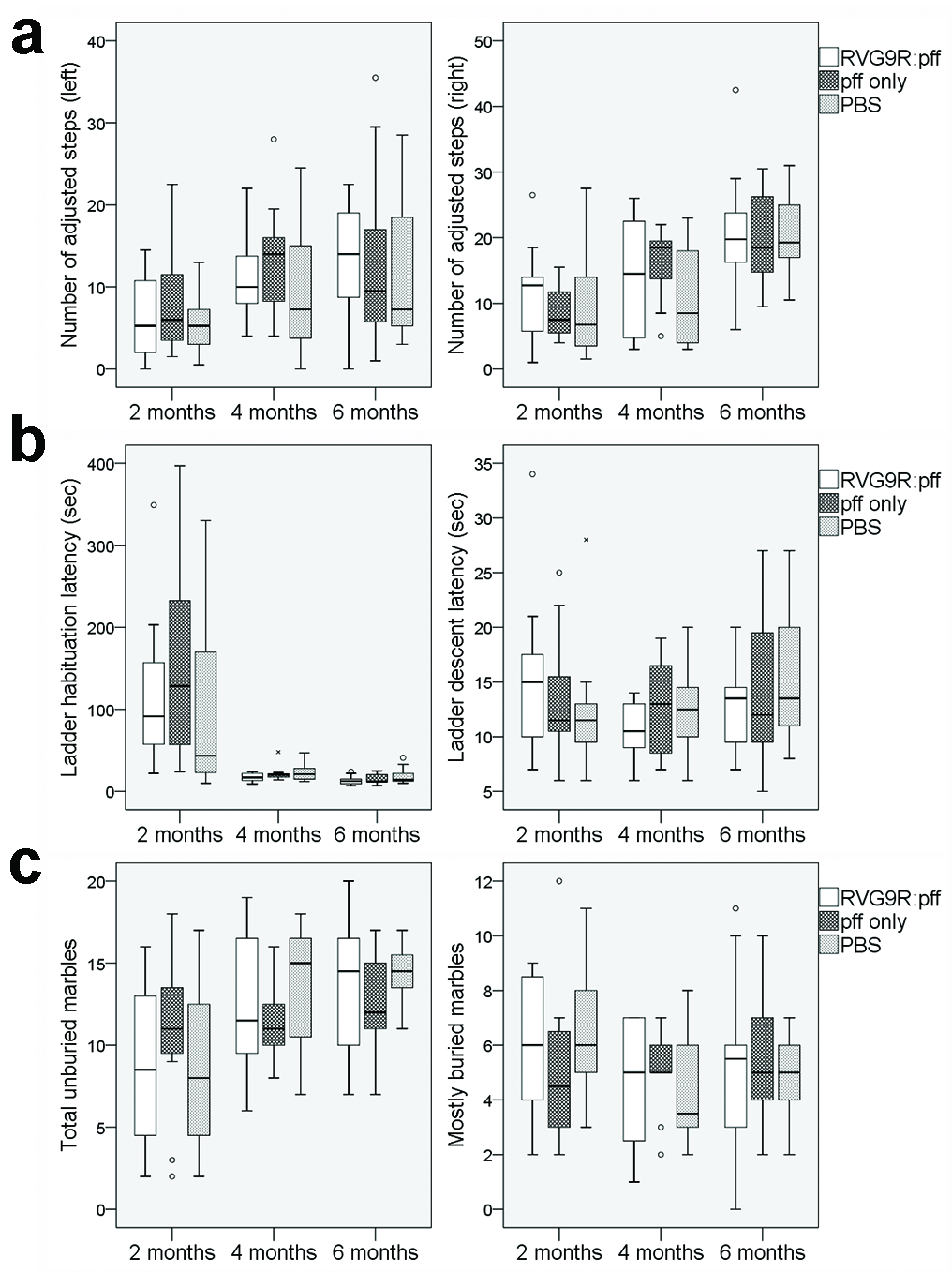
**

**Supplementary Figure 3: The effects of RVG9R:pff treatment in motor function.** There were no significant differences in the performance of locomotor activities (adjusted stepping test), gross motor coordination (ladder climbing test), and repetitive digging (marble burying test), between animals administered with RVG9R:pff or controls at any time points assessed. Data was analyzed using repeated measures ANOVA, *n*=12-24 per group per time point.

**
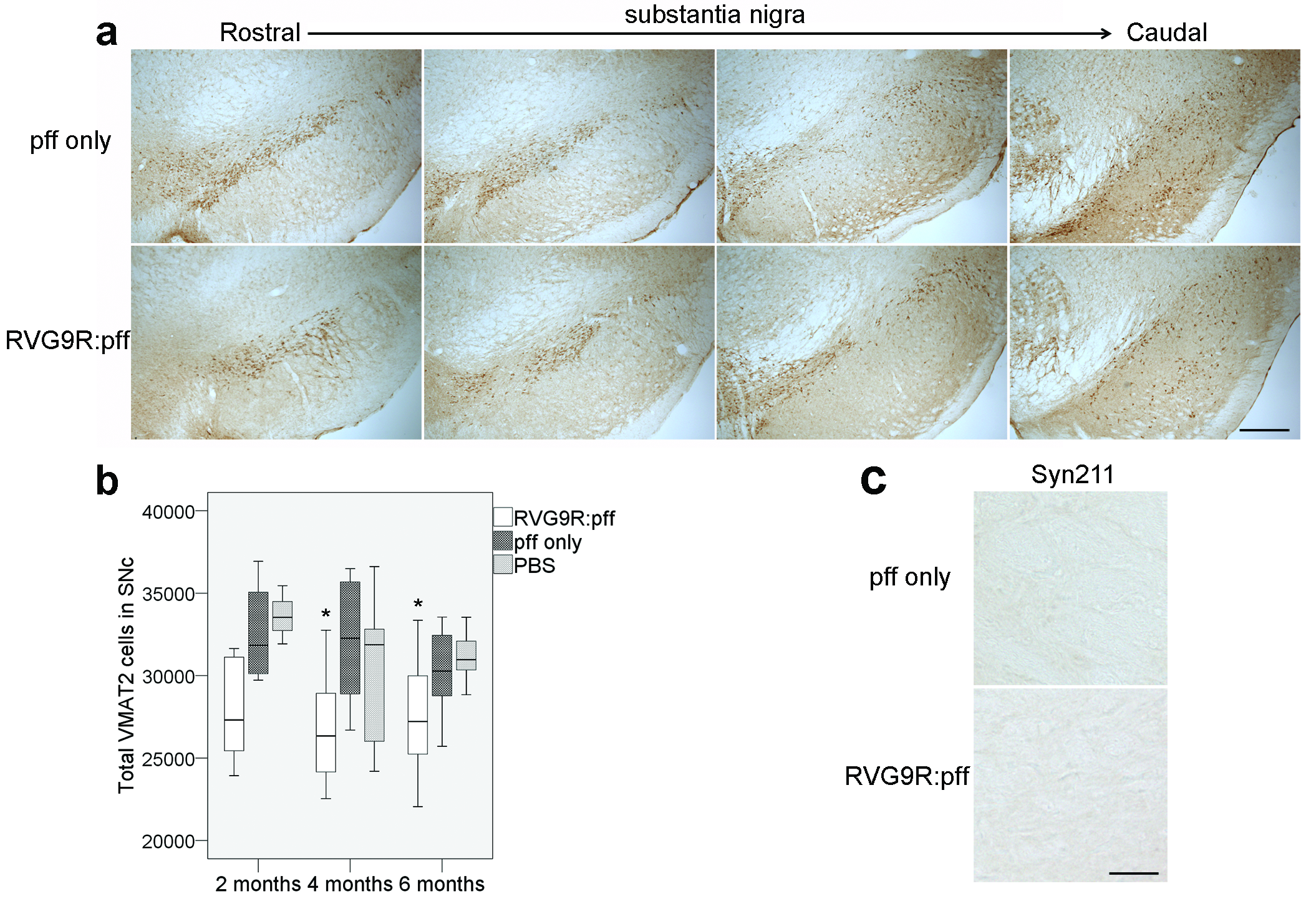
**

**Figure S4: RVG9R:pff induces dopaminergic degeneration, rather than down-regulation of dopaminergic phenotype, in the SNc.** To verify whether dopaminergic cell loss observed was due to a loss of TH expression as a result of α-synucleinopathy, the number of VMAT2^+^ neurons in the SNc was quantitated using unbiased stereology. (A) Representative images of the VMAT2^+^ neurons in the midbrain. (B) Similar to TH analysis, there was a significant treatment effect on VMAT2^+^ cell counts in the SNc (*F*_2,65_=15.874, *p*<0.001). Post-hoc analysis revealed that RVG9R:pff rats displayed significant dopaminergic neurodegeneration *vs* pff only (*p*=0.018) group at 4 months post-lesion, and between RVG9R:pff versus pff only (*p*=0.038) and PBS (*p*=0.04) groups at 6 months post-lesion. (C) The observed α-synucleinopathy in the brain of RVG9R:pff injected rats was due to endogenous α-synuclein aggregation, as no human α-synuclein could be observed in the CNS after 6 months post-injection. Data was analyzed using multivariate ANOVA, *n*=6-10 per group at 2- and 4-months post-injection, *n*=12 at 6 months post-injection (a-c). Scale bars, 500µm for (a), 100µm for (c).

**
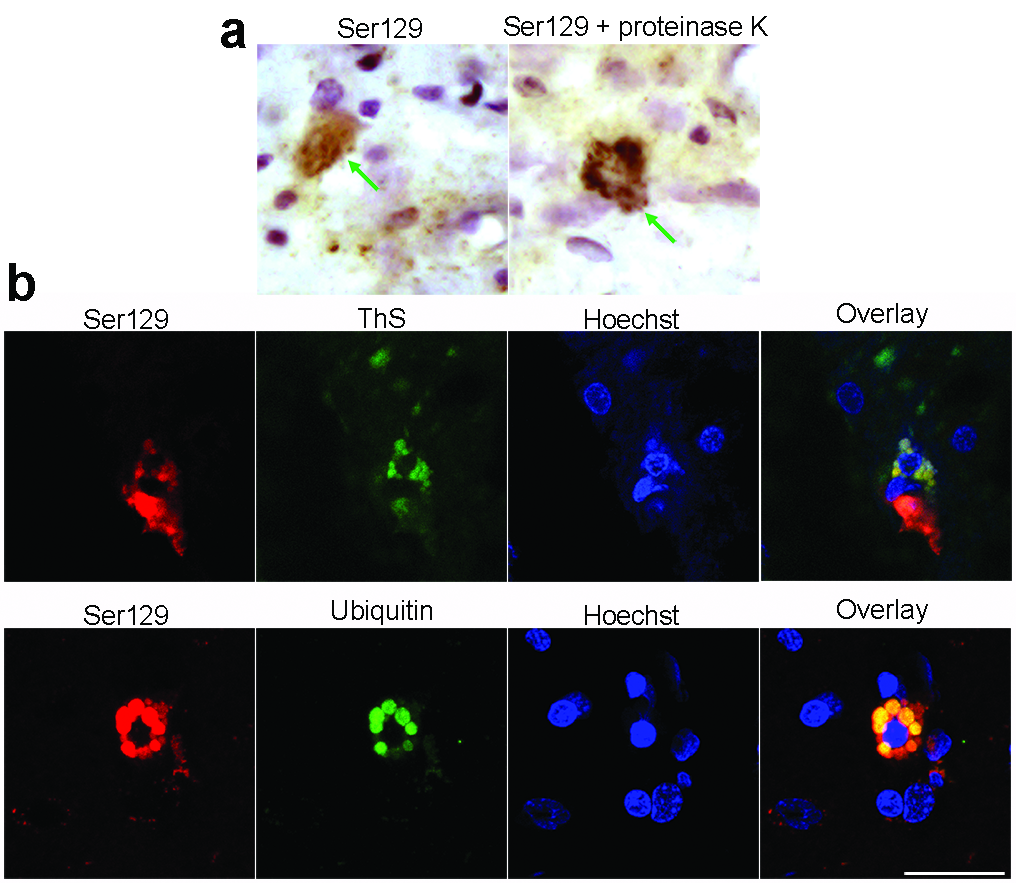
**

**Figure S5: RVG9R:pff induces LB-like inclusions in the brain stem.** (A) Representative DAB-peroxidase images of amorphous α-synuclein inclusions with or without proteinase-K digestion (green arrows). (B) These inclusions were both thioflavin-S positive and ubiquitylated, similar to those observed in clinical PD. *n*=12 per group; scale bars, 30µm.
